# *TP53* p.Arg337His germline mutation prevalence in Southern Brazil: Further evidence for mutation testing in young breast cancer patients

**DOI:** 10.1371/journal.pone.0209934

**Published:** 2018-12-31

**Authors:** Eriza Cristina Hahn, Camila Matzenbacher Bittar, Fernanda Sales Luis Vianna, Cristina Brinckmann Oliveira Netto, Jorge Villanova Biazús, Rodrigo Cericatto, José Antônio Cavalheiro, Márcia Portela de Melo, Carlos Henrique Menke, Eliane Rabin, Sandra Leistner-Segal, Patricia Ashton-Prolla

**Affiliations:** 1 Programa de Pós-Graduação em Genética e Biologia Molecular, Universidade Federal do Rio Grande do Sul (UFRGS), Porto Alegre, Brazil; 2 Laboratório de Medicina Genômica, Centro de Pesquisa Experimental do Hospital de Clínicas de Porto Alegre, Porto Alegre, Brazil; 3 Laboratório de Genética Molecular, Serviço de Genética Médica do HCPA, Porto Alegre, Brazil; 4 Serviço de Genética Médica do Hospital de Clínicas de Porto Alegre, Porto Alegre, Brazil; 5 Serviço de Mastologia do Hospital de Clínicas de Porto Alegre, Porto Alegre, Brazil; CNR, ITALY

## Abstract

Premenopausal breast cancer (BC) is a core tumor of Li-Fraumeni (LFS) and Li-Fraumeni-like (LFL) Syndromes, predisposition disorders caused by germline mutations in *TP53* gene. In the Southern and Southeastern regions of Brazil, a specific *TP53* germline mutation, c.1010G>A (p.Arg337His), was identified at a population frequency of 0.3%, the highest value ever described for a *TP53* germline variation. In Brazilian BC patients, carrier frequency can vary from 0.5% to 8.7%. The current study assessed carrier frequency by genotyping *TP53* c.1010G>A in 2 BC groups: 1) 315 patients unselected for age of diagnosis and family history (FH) and 2) 239 patients diagnosed before 46 years and without Chompret criteria for LFS or LFL. One carrier was identified in group 1 (0.3%; CI 95% 0.1–1.76%) and six carriers in group 2 (2.5%; CI 95% 0.93–5.39%). The frequencies differed significantly between groups (p = 0.04). The mutation carrier frequency observed in group 2 could justify mutation testing in BC patients diagnosed before 46 years and without Chompret criteria for LFS or LFL. Further studies in larger samples of BC patients of different ages and regions of the country are necessary to provide more definitive *TP53* p.Arg337His carrier frequencies in different scenarios.

## Introduction

Breast Cancer (BC) is the most commonly diagnosed cancer and the leading cause of death by cancer in women, with over 2 million new cases in 2018. In Brazil, BC incidence accounts for about 30% of all cancers diagnosed in women every year [1]. Most BC are sporadic and develop due to a combination of environmental and genetic risk factors, like low penetrance genetic variants with small effect [2,3]. However, about 10% of all BC cases, is thought to be inherited and caused by moderate or high penetrance germline mutations in cancer predisposition genes. Although the exact molecular alteration leading to Hereditary Breast Cancer (HBC) is still unknown in about half of these cases, germline mutations in several tumor suppressor genes have been identified in association with premenopausal breast cancer, including the *BRCA1*, *BRCA2* and *TP53*, among others [4].

*TP53* gene encodes the p53 protein, a transcription factor with a central role in the control and regulation of events related to the cell cycle, which is activated in response to several events, including DNA damage and hypoxia [5]. Approximately 50% of solid tumors have somatic mutations in *TP53*, which renders it the most frequently mutated gene in different types of cancers [6]. Germline mutations, on the other hand, are associated with Li-Fraumeni (LFS) and Li-Fraumeni-like (LFL) Syndromes, autosomal dominant disorders that predispose to multiple early-onset tumors, including premenopausal BC. In these syndromes, the estimated risk of developing at least one primary tumor by age 50 is around 80% and breast cancer is the most common solid tumor observed in adult women affected by these syndromes [7].

Germline mutations in *TP53* occur at a very low frequency in the general population (1:2000–5000 individuals) [8]. However, in the Southern and Southeastern of Brazil, one specific germline *TP53* mutation, c.1010G>A (p.Arg337His), also known as R337H, was identified at a population frequency of 0.3%, which is much higher than any other alteration described so far in this gene [9,10].

The first report of the p.Arg337His mutation in Brazil was in a cohort of children with apparently isolated adrenocortical carcinoma (ACC), in which 97% were carriers [11]. In 2007, the association of the mutation with LFS/LFL was confirmed in a report of families from different Brazilian states [12]. Moreover, the mutation was identified in patients with other solid tumors of the LFS/LFL spectrum, including choroid plexus carcinoma, breast cancer and sarcomas [13–15].

Prevalence studies of this germline mutation in different BC cohorts from Southern and Southeastern Brazil have described a wide range of carrier frequencies (0.5% to 8.7%) [14–20]. Identification of *TP53* p.Arg337His carriers is very important for cancer risk assessment, genetic counseling and definition of management in these individuals. Carriers are at an increased risk for multiple tumors at an early age, often in childhood and risk reducing intervention exist. In addition, a positive result in one individual prompts to the investigation of asymptomatic relatives, enabling proper risk management for those who carry the same variant. In this context, the aim of the present study was to estimate *TP53* p.Arg337His frequency in two groups of BC patients diagnosed in a public hospital in the State of Rio Grande do Sul (RS), in Southern Brazil, a region with high breast cancer incidence rates.

## Materials and methods

### Participants and study design

This study was approved by the institutional ethics committee of Hospital de Clínicas de Porto Alegre, Brazil which is recognized by the Office for Human Research Protections as an Institutional Review Board (IRB0000921). The patients included in this study were recruited from the Mastology and Medical Genetics Services of Hospital de Clínicas de Porto Alegre (HCPA), a public general and university hospital in the State of RS, Southern Brazil. Patients were recruited and analyzed in two separate groups. Patients of the first group were enrolled between 2002 and 2010 and the only inclusion criteria were a histologically proven diagnosis of breast cancer at or above age 18 years and willingness to participate in the study. At inclusion, patients were not selected regarding FH of cancer and hence, this group will be described as the “unselected” group or group 1. The second group analyzed, included 239 women diagnosed with breast cancer at or below age 45 years, who did not have Chompret criteria indicative for *TP53* mutation testing [21]. This group will be described as the “early-onset BC” group or group 2. The 45-year age limit was adopted to stratify groups in relation to age at BC diagnosis, since this cutoff is commonly used in LFS studies and also in guidelines that indicate *TP53* mutation testing (i.e. National Comprehensive Cancer Network–NCCN, Chompret criteria) [21]. The study was approved by the institutional ethics committee of Hospital de Clínicas de Porto Alegre, Brazil and all patients provided written informed consent (protocol numbers 2004–0438 and 2014–0658). Demographic, clinical and family history data were obtained by review of medical records.

### Genotyping

Genomic DNA was obtained from the leukocyte fraction of peripheral blood, using the Salting-out methodology or the commercial kit Flexigene (Qiagen) [22]. In one of the mutation carriers (unselected group) tumor samples were available and loss of heterozigosity (LOH) was evaluated in DNA from the breast tumor. To this end, a pathologist prepared and analyzed HE slides containing slices of the tumor sample. Next, a BC region was delineated, microdissected and used for DNA extraction with the commercial kit ReliaPrep FFPE gDNA Miniprep System (Promega).

Germline mutation screening and tumor tissue analysis were performed by allelic discrimination using a TaqMan assay, with customized probes for the wild-type (c.1010G) and mutant (c.1010A) alleles. In all samples with a mutant allele, genotyping was confirmed by Sanger sequencing, as described by Giacomazzi et al. 2014. Assessment of the haplotype associated with the mutant allele was done as described by Garritano et al 2010.

### Statistical analysis

The mutation frequency was obtained by simple counting of the number of patients with at least one mutated allele. Clinical and family history data of the two groups were compared by Chi-Square Test. Confidence Intervals of the mutation frequency were estimated using WinPepi Software (version 11.65). Comparisons of mutation prevalence data between groups was done using Fisher’s Exact Test. Values of p<0.05 were considered as a statically significant result.

## Results

### Demographic and clinical data

Demographic and clinical data of the two groups of patients studied are shown in Table 1. In both groups, women account for more than 99% of the sample. Mean age at BC diagnosis was 56.3 years (SD: 13.1 years, range: 28–89 years) in group 1 and 36.7 years (SD: 5.3 years, range: 23–45 years). In group 1, most patients were diagnosed after age 45 years (74.9%) and, regarding FH of cancer, 62.2% of the participants reported a positive FH of cancer, considering report of at least one 1^st^, 2^nd^ or 3^rd^-degree relative with cancer, without, necessarily, being compatible with a history of predisposition to hereditary cancer syndromes or fulfilling the established clinical criteria for *TP53* mutation testing (Table 1). The most common histological BC type was invasive ductal carcinoma (72.4%).

**Table 1:** Demographic and clinical data of the “unselect group”–group 1 and “early- onset BC”–group 2).

	Group 1		Group 2		p (chi-square test)
	**N**	**%**	**N**	**%**	
**Gender**					
*Female*	313	99.3	237	99.2	0.78
*Male*	2	0.7	2	0.8	
**Age at breast cancer diagnosis (years)**					
*≤45*	70	22.2	239	100.0	
*≥46*	236	74.9	-	-	
*Not specified*	9	2.9	-	-	
*Mean*, *SD*	56.3, 13.1 years		36.6, 5.3 years		**<0.0001 (t test)**
**Additional primary tumors**					
*Yes*	34	10.8	23	9.6	0.429
*No*	255	81.0	216	90.4	
*Not specified*	26	8.2	-		
**Personal history of cancer (other tumors)**					
*Breast*	14	41.1	16	69.7	0.106
*Endometrial*	9	26.5	1	4.3	
*Colorectal*	2	5.9	1	4.3	
*Others*	9	26.5	5	21.7	
**Family History of Breast Cancer**					
*Yes*	95	13.7	139	58.2	**<0.001**
*No*	177	30.1	95	39.7	
*Not specified*	43	56.2	5	2.1	
***TP53* p.Arg337His prevalence**	1	0.32	6	2.51	0.04**

SD: Standard Deviation

*p<0.05

** Fisher´s exact test

*TP53* p.Arg337His frequency in the unselected group was 0.3% (1/315–95% CI: 0.01–1.76%), equivalent to that observed in the general population of the Southern and Southeastern regions of Brazil. The only carrier identified was from a small city from the State of São Paulo, reported Portuguese ancestry, positive family history of cancer and showed the same haplotype previously described as founder haplotype [23]. She was diagnosed with an invasive ductal carcinoma of the breast (hormone receptor negative, HER2-positive, overexpressing p53) at age 67 years. At age 69 years, the proband was diagnosed with a gastric adenocarcinoma, and died shortly thereafter. Genotyping of the breast tumor did not reveal loss of heterozygosity in the tumor.

In the second group of patients analyzed, 6 carriers were identified (6/239; 2.5%, 95% CI: 0.93–5.38%), all with the founder haplotype. Tumors samples were not available for mutation testing and assessment of LOH. The carrier frequencies between groups differed significantly (p = 0.04).

## Discussion

By now, several studies have provided evidence that the germline *TP53* mutation p.Arg337His is present at an exceedingly high frequency in the general population from Southern and Southeastern Brazil. Although penetrance of this oligomerization domain mutant seems to be decreased in relation to DNA-binding domain mutations, its populational frequency is unsurpassed by any other cancer predisposing germline mutation [9, 10, 24]. Carrier families have different phenotypes, showing either very few or simplex cancer cases or more complex family histories ranging from phenotypes not clearly suggestive of LFS/LFL to phenotypes typical of these syndromes. In p.Arg337His families, breast cancer is the most common solid tumor diagnosed in adult carriers and its diagnosis can occur at different ages [9, 15, 24].

The understanding of genetic factors related to BC is essential, because its incidence is increasing worldwide, with varying mortality rates: developed countries tend to present a reduction, while developing countries show increasing rates, a trend also observed in Southern Brazil [25,26]. In Porto Alegre, the capital that proportionally shows the highest BC incidence rate in the country, an important proportion of women is hospitalized or die from BC before 50 years of age [27,28]. A population-based study carried out in the city of Porto Alegre with more than 9,000 participants, revealed that 6.2% of women visiting primary health care units presented a phenotype compatible with HBC, and of these, approximately 70% met criteria for LFS or LFL [29]. From these numbers, one could hypothesize that the *TP53* p.Arg337His mutation is an important contributor to the BC-related burden in the region.

In BC, age at diagnosis is an important point to consider. In LFS and LFL, several studies have described a higher mutation prevalence among women with pre-menopausal when compared to post-menopausal breast cancer and phenotypic criteria that suggest the syndrome (i.e. Chompret criteria) use the age of 45 years as a cutoff age for breast cancer diagnosis [15]. In the current study, we have analyzed two groups of patients. In the unselected group of women with breast cancer, recruited regardless of age at cancer diagnosis and cancer family history, carrier frequency was equal to that observed in the general population. This was quite surprising, since one would expect a slightly increased prevalence. However, a previous study comprising 390 BC patients–that were also not selected regarding FH—revealed a similar prevalence to that seen in the unselected group of the present study, 0.5% [18]. In this group, mean age at BC diagnosis (56.3 years), and the proportion of women diagnosed after 45 years (almost 75%), may have influenced mutation prevalence. Carrier frequency in the second group of patients (diagnosed at or below 45 years who were recruited from a cancer genetics clinic and did not have criteria for *TP53* mutation testing) was 2.5% similar to mutation prevalences observed in 2 previous studies that included women with a FH compatible with hereditary breast and ovarian cancer syndrome, 0.94% and 2.5% [19, 20]. Taken together, these findings suggest that a BC diagnosis below age 46 years is an important indicator of *TP53* p.Arg337His presence.

Provided that the carrier frequency among women with breast cancer <46 years without a family history is 2.5%, the next question is whether this prevalence justifies routine mutation testing in such cases. In the Ashkenazi Jewish population, where founder *BRCA1* and *BRCA2* mutations are present in about 2.5% of the individuals, cost-effectiveness studies have implied that population testing is justified [30]. On the other hand, for more complex and admixed populations, where no *BRCA* founder mutations exist and mutation prevalence is around 0.25%, several authors have stated that population testing is not cost-effective and therefore not justified [31,32].

Additional studies with larger samples of patients should be undertaken to provide definitive carrier frequencies in different clinical scenarios. However, the present study, provides further arguments to suggest a role for mutation testing of all women diagnosed with BC below age 46 years in Southern Brazil. A better understanding of the penetrance and the cost-effectiveness of *TP53* p.Arg337His testing in women with pre-menopausal breast cancer regardless of family history criteria could contribute to determine if such testing is justified from a public health care perspective.

## Supporting information

S1 Table Epidemiological data collection of the study participants. (PDF)
