# Supplementary material for: TP53 p.Arg337His germline mutation prevalence in Southern Brazil: Further evidence for mutation testing in young breast cancer patients
Source: PLoS One. 2018 Dec 31;13(12):e0209934. doi: 10.1371/journal.pone.0209934 (PMC6312227; doi:10.1371/journal.pone.0209934)
Supplement: S1 Table — (PDF) [file pone.0209934.s001.pdf]

Table S1: Epidemiological data collection of the study participants.

Group	ID	Gender	Age at BC diagnosis (years)	Additional primary tumors	Personal history of cancer (other tumors)	Family history of BC	*TP53* p.Arg337His
1	1	F	54	S	BREAST	NS	N
1	2	F	32	NS		NS	N
1	3	F	55	N		N	N
1	4	F	51	NS		NS	N
1	5	F	47	N		NS	N
1	6	F	76	N		NS	N
1	7	F	45	S	BREAST	NS	N
1	8	F	47	N		Y	N
1	9	F	57	N		N	N
1	10	F	44	N		N	N
1	11	F	31	NS		Y	N
1	12	F	52	NS		N	N
1	13	F	65	NS		N	N
1	14	F	28	NS		N	N
1	15	F	77	NS		N	N
1	16	F	61	NS		NS	N
1	17	F	46	NS		NS	N
1	18	F	44	N		N	N
1	19	F	51	N		N	N
1	20	F	43	NS		NS	N
1	21	F	66	N		NS	N
1	22	F	33	S	LUNG	NS	N
1	23	F	68	N		NS	N

1	24	F	53	N		N	N
1	25	F	45	NS		NS	N
1	26	F	28	S	BREAST	N	N
1	27	F	31	S	BREAST	Y	N
1	28	F	61	N		N	N
1	29	F	52	N		Y	N
1	30	F	39	N		Y	N
1	31	F	60	N		N	N
1	32	F	45	N		N	N
1	33	F	51	N		Y	N
1	34	F	59	N		N	N
1	35	F	68	N		NS	N
1	36	F	64	N		NS	N
1	37	F	62	N		N	N
1	38	F	78	N		N	N
1	39	F	37	N		NS	N
1	40	F	49	N		N	N
1	41	F	65	N		N	N
1	42	F	37	N		Y	N
1	43	F	56	N		N	N
1	44	F	52	N		Y	N
1	45	F	64	N		N	N
1	46	F	60	S	SKIN	Y	N
1	47	F	49	N		Y	N
1	48	F	40	S	BREAST	N	N
1	49	F	80	N		N	N
1	50	F	69	N		NS	N
1	51	F	43	N		N	N
1	52	F	59	N		N	N
1	53	F	60	N		N	N
1	54	F	50	N		Y	N
1	55	F	72	N		Y	N
1	56	F	85	NS		NS	N

1	57	F	43	N		Y	N
1	58	F	44	N		Y	N
1	59	F	65	N		N	N
1	60	F	45	N		N	N
1	61	F	84	N		N	N
1	62	F	54	N		N	N
1	63	F	49	N		N	N
1	64	F	63	N		Y	N
1	65	F	47	S	BREAST	Y	N
1	66	F	53	N		N	N
1	67	F	NS	N		Y	N
1	68	F	51	N		Y	N
1	69	F	51	S	ENDOMETRIAL	N	N
1	70	F	45	S	HODGKIN	NS	N
1	71	F	NS	N		N	N
1	72	F	53	N		Y	N
1	73	F	46	N		Y	N
1	74	F	76	N		N	N
1	75	F	43	N		N	N
1	76	F	44	N		N	N
1	77	F	75	N		N	N
1	78	F	60	S	ENDOMETRIAL	N	N
1	79	F	54	N		N	N
1	80	F	53	N		N	N
1	81	F	45	N		N	N
1	82	F	49	N		Y	N
1	83	F	73	N		Y	N
1	84	F	72	N		N	N
1	85	F	61	S	BREAST	Y	N
1	86	F	38	N		Y	N
1	87	F	45	N		N	N
1	88	F	77	N		N	N
1	89	F	54	N		N	N
1	90	F	57	N		N	N

1	91	F	61	N		Y	N
1	92	F	76	S	ENDOMETRIAL	Y	N
1	93	F	66	N		N	N
1	94	F	59	N		N	N
1	95	F	46	N		N	N
1	96	F	51	N		N	N
1	97	F	56	N		N	N
1	98	F	67	S	STOMACH	N	P
1	99	F	55	N		Y	N
1	100	F	31	N		N	N
1	101	F	74	N		Y	N
1	102	F	64	N		N	N
1	103	F	50	N		Y	N
1	104	F	52	N		Y	N
1	105	F	50	N		N	N
1	106	F	37	S	BREAST	Y	N
1	107	F	40	N		N	N
1	108	F	33	N		N	N
1	109	F	46	N		N	N
1	110	F	50	N		NS	N
1	111	F	39	N		N	N
1	112	F	77	N		N	N
1	113	F	41	N		Y	N
1	114	F	66	N		N	N
1	115	F	71	N		Y	N
1	116	F	57	N		N	N
1	117	F	41	N		NS	N
1	118	F	67	N		N	N
1	119	F	71	S	BREAST	Y	N
1	120	F	38	S	BREAST	Y	N
1	121	F	56	N		N	N
1	122	F	62	S	BREAST	N	N
1	123	M	35	N		Y	N
1	124	F	56	N		N	N

1	125	F	51	N		N	N
1	126	F	71	N		Y	N
1	127	F	62	S	SKIN	Y	N
1	128	F	54	N		Y	N
1	129	F	42	N		N	N
1	130	F	53	N		Y	N
1	131	F	50	N		N	N
1	132	F	60	N		Y	N
1	133	F	36	N		Y	N
1	134	F	63	NS		Y	N
1	135	F	58	N		N	N
1	136	F	54	N		N	N
1	137	M	66	N		N	N
1	138	F	66	N		N	N
1	139	F	70	N		N	N
1	140	F	62	N		N	N
1	141	F	37	N		Y	N
1	142	F	31	N		N	N
1	143	F	32	N		N	N
1	144	F	77	S	SKIN	N	N
1	145	F	45	N		N	N
1	146	F	33	N		N	N
1	147	F	73	N		Y	N
1	148	F	69	N		N	N
1	149	F	73	N		N	N
1	150	F	73	N		Y	N
1	151	F	73	N		N	N
1	152	F	71	S	ENDOMETRIAL	NS	N
1	153	F	57	N		N	N
1	154	F	NS	NS		NS	N
1	155	F	77	S	VULVA	N	N
1	156	F	50	N		N	N
1	157	F	47	N		N	N
1	158	F	41	N		N	N

1	159	F	40	N		Y	N
1	160	F	55	N		N	N
1	161	F	68	N		NS	N
1	162	F	53	N		N	N
1	163	F	51	N		N	N
1	164	F	64	N		NS	N
1	165	F	58	N		N	N
1	166	F	28	N		N	N
1	167	F	59	N		Y	N
1	168	F	48	NS		N	N
1	169	F	41	NS		Y	N
1	170	F	69	N		NS	N
1	171	F	67	N		Y	N
1	172	F	51	N		N	N
1	173	F	44	N		NS	N
1	174	F	42	N		N	N
1	175	F	52	N		NS	N
1	176	F	49	NS		NS	N
1	177	F	60	N		NS	N
1	178	F	55	N		N	N
1	179	F	77	N		N	N
1	180	F	69	S	ENDOMETRIAL	N	N
1	181	F	74	N		N	N
1	182	F	74	N		N	N
1	183	F	78	N		Y	N
1	184	F	45	N		N	N
1	185	F	75	S	ENDOMETRIAL	N	N
1	186	F	NS	N		NS	N
1	187	F	31	N		N	N
1	188	F	62	N		N	N
1	189	F	65	N		N	N
1	190	F	60	N		N	N
1	191	F	45	N		Y	N
1	192	F	56	N		N	N

1	193	F	45	N		N	N
1	194	F	45	N		N	N
1	195	F	72	S	BLADDER	Y	N
1	196	F	52	N		N	N
1	197	F	51	N		N	N
1	198	F	76	N		N	N
1	199	F	58	N		Y	N
1	200	F	46	N		N	N
1	201	F	67	N		Y	N
1	202	F	48	N		Y	N
1	203	F	73	N		N	N
1	204	F	43	N		NS	N
1	205	F	50	N		Y	N
1	206	F	40	N		N	N
1	207	F	64	N		N	N
1	208	F	68	N		N	N
1	209	F	55	NS		N	N
1	210	F	62	N		N	N
1	211	F	42	N		N	N
1	212	F	50	N		N	N
1	213	F	51	S	ENDOMETRIAL	Y	N
1	214	F	52	N		Y	N
1	215	F	45	N		NS	N
1	216	F	69	N		Y	N
1	217	F	37	N		N	N
1	218	F	70	N		N	N
1	219	F	74	N		NS	N
1	220	F	56	N		Y	N
1	221	F	NS	NS		NS	N
1	222	F	62	N		Y	N
1	223	F	45	N		N	N
1	224	F	54	NS		NS	N
1	225	F	79	NS		N	N
1	226	F	NS	N		NS	N

1	227	F	52	N		N	N
1	228	F	62	N		N	N
1	229	F	65	N		Y	N
1	230	F	70	N		Y	N
1	231	F	72	N		Y	N
1	232	F	55	N		N	N
1	233	F	55	N		Y	N
1	234	F	49	N		N	N
1	235	F	81	N		N	N
1	236	F	57	N		Y	N
1	237	F	55	S	BREAST	N	N
1	238	F	79	N		N	N
1	239	F	62	NS		NS	N
1	240	F	52	N		N	N
1	241	F	63	N		Y	N
1	242	F	48	N		Y	N
1	243	F	57	N		N	N
1	244	F	57	N		Y	N
1	245	F	68	N		N	N
1	246	F	66	N		N	N
1	247	F	74	N		Y	N
1	248	F	53	N		N	N
1	249	F	54	N		Y	N
1	250	F	62	N		N	N
1	251	F	60	S	HODGKIN; BREAST	N	N
1	252	F	72	N		N	N
1	253	F	60	S	COLORECTAL	N	N
1	254	F	54	N		N	N
1	255	F	63	N		Y	N
1	256	F	70	S	COLORECTAL	N	N
1	257	F	46	N		Y	N
1	258	F	46	N		Y	N
1	259	F	NS	NS		NS	N
1	260	F	62	N		N	N

1	261	F	49	N		N	N
1	262	F	46	N		N	N
1	263	F	52	N		NS	N
1	264	F	55	N		N	N
1	265	F	42	N		N	N
1	266	F	66	N		N	N
1	267	F	45	NS		Y	N
1	268	F	50	N		Y	N
1	269	F	86	N		N	N
1	270	F	85	N		N	N
1	271	F	85	N		Y	N
1	272	F	34	N		Y	N
1	273	F	48	S	MUSCLE	N	N
1	274	F	64	N		N	N
1	275	F	57	N		N	N
1	276	F	50	N		Y	N
1	277	F	46	N		N	N
1	278	F	57	N		N	N
1	279	F	64	N		N	N
1	280	F	79	N		N	N
1	281	F	29	NS		Y	N
1	282	F	60	N		Y	N
1	283	F	38	N		Y	N
1	284	F	47	N		N	N
1	285	F	62	N		Y	N
1	286	F	56	N		N	N
1	287	F	73	N		N	N
1	288	F	43	N		N	N
1	289	F	78	N		NS	N
1	290	F	76	N		Y	N
1	291	F	62	S	ENDOMETRIAL	Y	N
1	292	F	70	N		N	N
1	293	F	71	N		Y	N
1	294	F	79	N		Y	N

1	295	F	67	N		N	N
1	296	F	NS	N		NS	N
1	297	F	46	N		Y	N
1	298	F	56	N		N	N
1	299	F	NS	NS		NS	N
1	300	F	89	N		N	N
1	301	F	49	N		N	N
1	302	F	36	N		N	N
1	303	F	86	N		N	N
1	304	F	64	S	BREAST	N	N
1	305	F	64	N		N	N
1	306	F	63	N		N	N
1	307	F	57	N		Y	N
1	308	F	44	N		Y	N
1	309	F	58	N		Y	N
1	310	F	57	N		Y	N
1	311	F	44	N		N	N
1	312	F	70	N		N	N
1	313	F	37	N		N	N
1	314	F	40	S	ENDOMETRIAL	Y	N
1	315	F	49	N		Y	N
2	316	F	33	Y	ENDOMETRIAL	N	N
2	317	F	23	N		Y	P
2	318	F	32	Y	COLORECTAL	N	N
2	319	F	23	N		N	N
2	320	F	24	N		N	N
2	321	F	24	N		N	N
2	322	F	24	N		Y	N
2	323	F	26	N		N	N
2	324	F	26	N		N	N
2	325	F	26	N		Y	N
2	326	F	27	Y	BREAST	Y	N
2	327	F	27	N		N	N
2	328	F	27	N		N	N

2	329	F	28	N		N	N
2	330	F	28	N		N	N
2	331	F	28	N		Y	N
2	332	F	28	N		Y	N
2	333	F	29	N		Y	N
2	334	F	29	Y	BREAST	N	N
2	335	F	29	N		Y	N
2	336	F	29	Y	BREAST	Y	N
2	337	F	29	N		Y	N
2	338	F	29	N		N	N
2	339	F	30	N		Y	N
2	340	F	30	N		Y	N
2	341	F	30	N		Y	N
2	342	F	30	N		N	N
2	343	F	30	N		N	N
2	344	F	30	N		Y	N
2	345	F	30	N		Y	N
2	346	F	30	N		Y	N
2	347	F	30	N		Y	N
2	348	F	30	N		Y	N
2	349	F	31	N		Y	N
2	350	F	31	N		Y	N
2	351	F	31	N		N	N
2	352	F	31	N		N	N
2	353	F	31	N		Y	P
2	354	F	31	N		N	N
2	355	F	31	N		N	N
2	356	F	31	N		Y	N
2	357	F	32	N		Y	N
2	358	F	32	N		Y	N
2	359	F	33	Y	BREAST	Y	N
2	360	F	32	N		N	N
2	361	F	32	N		Y	N
2	362	F	32	N		N	N

2	363	F	32	N		N	N
2	364	F	32	N		Y	N
2	365	F	32	N		Y	N
2	366	F	32	N		N	N
2	367	F	32	N		N	N
2	368	F	32	N		Y	N
2	369	F	32	N		Y	N
2	370	F	33	N		Y	N
2	371	F	33	N		N	N
2	372	F	33	N		N	N
2	373	F	33	N		Y	N
2	374	F	33	N		Y	N
2	375	F	33	N		N	N
2	376	F	33	N		N	N
2	377	F	34	Y	BREAST	N	N
2	378	F	33	N		N	N
2	379	F	33	N		Y	N
2	380	F	33	N		N	N
2	381	F	33	N		Y	P
2	382	F	35	Y	BREAST	Y	N
2	383	F	34	N		Y	N
2	384	F	34	N		N	N
2	385	F	34	N		N	N
2	386	F	34	N		Y	N
2	387	F	34	N		Y	N
2	388	F	35	Y	BREAST	Y	N
2	389	F	34	N		Y	N
2	390	F	34	N		N	N
2	391	F	34	N		Y	N
2	392	F	34	N		Y	N
2	393	F	34	N		Y	N
2	394	F	34	N		N	N
2	395	F	35	N		N	N
2	396	F	35	Y	BREAST	Y	N

2	397	F	35	N		Y	N
2	398	F	35	N		Y	N
2	399	F	35	N		Y	N
2	400	F	35	N		N	N
2	401	F	37	Y	BREAST	Y	N
2	402	F	37	Y	BREAST	Y	N
2	403	F	35	N		N	N
2	404	F	35	N		N	N
2	405	F	35	N		N	N
2	406	F	35	N		Y	N
2	407	F	35	N		N	N
2	408	F	39	Y	BREAST	Y	N
2	409	F	35	N		N	N
2	410	F	35	N		N	N
2	411	F	35	N		Y	N
2	412	F	35	N		Y	N
2	413	F	35	N		N	N
2	414	F	35	N		N	N
2	415	F	36	N		Y	N
2	416	F	36	N		Y	N
2	417	F	36	N		Y	N
2	418	F	36	N		N	N
2	419	F	36	N		N	N
2	420	F	36	N		Y	N
2	421	F	36	N		Y	N
2	422	F	36	N		Y	N
2	423	F	36	N		Y	N
2	424	F	36	N		Y	N
2	425	F	37	N		Y	N
2	426	F	37	N		Y	N
2	427	F	37	N		Y	N
2	428	F	37	N		N	N
2	429	F	37	N		N	N
2	430	F	39	Y	BREAST	Y	N

2	431	F	42	Y	BREAST	N	N
2	432	F	37	N		Y	N
2	433	F	37	N		N	N
2	434	F	37	N		Y	N
2	435	F	37	N		N	N
2	436	F	37	N		N	N
2	437	F	37	N		Y	N
2	438	F	37	N		Y	N
2	439	F	37	N		N	N
2	440	F	37	N		N	N
2	441	F	38	N		N	N
2	442	F	38	N		Y	N
2	443	M	38	N		Y	N
2	444	F	38	N		Y	N
2	445	F	38	N		Y	N
2	446	F	38	N		N	N
2	447	F	38	N		Y	N
2	448	F	38	N		N	N
2	449	F	38	N		Y	N
2	450	F	38	N		N	N
2	451	F	38	N		Y	N
2	452	F	38	N		NS	N
2	453	F	38	N		Y	N
2	454	F	38	N		Y	N
2	455	F	38	N		Y	N
2	456	F	39	N		N	N
2	457	F	39	N		Y	N
2	458	F	39	N		Y	N
2	459	F	39	N		Y	N
2	460	F	39	N		Y	N
2	461	F	39	N		NS	N
2	462	F	39	N		Y	N
2	463	F	39	N		N	N
2	464	F	39	N		Y	N

2	465	F	39	N		Y	N
2	466	F	39	N		N	N
2	467	F	39	N		Y	N
2	468	F	39	N		Y	N
2	469	F	39	N		Y	N
2	470	F	39	N		N	N
2	471	F	39	N		Y	N
2	472	F	43	Y	BREAST	N	N
2	473	F	44	Y	BREAST	Y	N
2	474	F	40	N		Y	N
2	475	F	40	N		Y	N
2	476	F	40	N		Y	N
2	477	F	40	N		N	N
2	478	F	40	N		Y	N
2	479	F	40	N		Y	N
2	480	F	40	N		Y	N
2	481	F	40	N		Y	P
2	482	F	40	N		Y	N
2	483	F	40	N		Y	N
2	484	F	40	N		Y	N
2	485	M	41	N		N	N
2	486	F	41	N		N	N
2	487	F	41	N		N	N
2	488	F	41	N		Y	N
2	489	F	41	N		Y	N
2	490	F	41	N		N	N
2	491	F	41	N		N	N
2	492	F	41	N		Y	N
2	493	F	41	N		N	N
2	494	F	41	N		Y	N
2	495	F	41	N		NS	N
2	496	F	41	N		Y	N
2	497	F	41	N		Y	N
2	498	F	41	N		N	N

2	499	F	41	N		Y	N
2	500	F	41	N		NS	N
2	501	F	41	N		N	N
2	502	F	41	N		N	N
2	503	F	43	Y	SKIN	NS	N
2	504	F	42	N		Y	N
2	505	F	42	N		N	N
2	506	F	42	N		Y	N
2	507	F	42	N		Y	N
2	508	F	42	N		Y	N
2	509	F	42	Y	OVARY	Y	N
2	510	F	42	N		N	N
2	511	F	42	N		N	N
2	512	F	42	N		Y	N
2	513	F	42	N		Y	N
2	514	F	35	Y	OVARY	Y	N
2	515	F	42	N		Y	N
2	516	F	42	Y	PANCREAS	Y	P
2	517	F	42	N		Y	N
2	518	F	43	N		Y	N
2	519	F	43	N		N	N
2	520	F	43	N		Y	N
2	521	F	43	N		N	N
2	522	F	43	N		Y	N
2	523	F	23	Y	PAROTID	N	N
2	524	F	43	N		N	N
2	525	F	23	Y	BREAST	Y	N
2	526	F	43	N		N	N
2	527	F	43	N		Y	N
2	528	F	43	N		N	N
2	529	F	43	N		Y	N
2	530	F	43	N		Y	P
2	531	F	43	N		Y	N
2	532	F	43	N		N	N

2	533	F	44	N		N	N
2	534	F	42	N		N	N
2	535	F	44	N		Y	N
2	536	F	44	N		N	N
2	537	F	44	N		N	N
2	538	F	44	N		N	N
2	539	F	44	N		Y	N
2	540	F	44	N		Y	N
2	541	F	44	N		N	N
2	542	F	44	N		N	N
2	543	F	44	N		Y	N
2	544	F	44	N		Y	N
2	545	F	45	N		N	N
2	546	F	45	N		N	N
2	547	F	45	N		N	N
2	548	F	45	N		Y	N
2	549	F	45	N		Y	N
2	550	F	45	N		Y	N
2	551	F	45	N		Y	N
2	552	F	45	N		N	N
2	553	F	45	N		Y	N
2	554	F	37	N		Y	N

Table legend:
ID: participant identification number
1: group 1 (patients unselected for age of diagnosis and family history).
2: group 2 (patients diagnosed before 46 years and without Chompret criteria for Li-Fraumeni or Li-Fraumeni-like Syndromes).
F: female
M: Male
N: No
Y: Yes
NS: Not specified
BC: Breast Cancer
